# Mice with low levels of Shc proteins display reduced glycolytic and increased gluconeogenic activities in liver

**DOI:** 10.1016/j.bbrep.2016.06.021

**Published:** 2016-06-30

**Authors:** Kevork Hagopian, Kyoungmi Kim, José Alberto López-Dominguez, Alexey A. Tomilov, Gino A. Cortopassi, Jon J. Ramsey

**Affiliations:** a VM Molecular Biosciences, School of Veterinary Medicine, University of California, Davis 1089 Veterinary Medicine Dr, VM3B, Davis, CA 95616, USA; b Department of Public Health Sciences, University of California Davis, Davis, CA 95616, USA

**Keywords:** Gly, glycogen, Glu, glucose, G6P, glucose-6-phosphate, F6P, fructose-6phosphate, F1, 6BP, fructose-1,6-bisphosphate, PEP, phosphoenolpyruvate, Pyr, pyruvate, Lac, lactate, Cit, citrate, Mal, malate, Glut, glutamate, α-KG, α-ketoglutarate, Ac-CoA, acetyl-CoA, CoA, coenzyme A, F2, 6BP, fructose-2,6-bisphosphate, Fru, fructose, F1P, fructose-1-phosphate, GK, glucokinase, PFK1, phosphofructokinase-1, PK, pyruvate kinase, PC, pyruvate carboxylase, PEPCK, phosphenolpyruvate carboxykinase, FBPase, fructose1,6-bispohosphatase, G6Pase, glucose-6-phosphatase, ALT, alanine transaminase, AST, aspartate transaminase, TRT, tryptophan transaminase, TYT, tyrosine transaminase, GKRP, glucokinase regulatory protein, AMPK, AMP-activated protein kinase, FOXO1, Forkhead Box O1 protein, Shc proteins, Fasting, Glycolysis, Gluconeogenesis, Metabolism, Fuel selection

## Abstract

Shc proteins play a role in energy metabolism through interaction with the insulin receptor. The aim of this study was to determine whether Shc proteins influence liver glycolysis and gluconeogenesis under both fed and fasted states. Decreased glycolytic and increased gluconeogenic and transamination enzyme activities were observed in ShcKO versus WT mice. Levels of key regulatory metabolites, such as fructose-2,6-bisphosphate, matched the activity of metabolic pathways. Protein levels of glycolytic and gluconeogenic enzymes were not different. pAMPK protein levels increased with fasting and were higher in ShcKO versus WT mice. Therefore, Shc proteins play a role in shifting the metabolism from glucose oxidation to gluconeogenesis and lipid catabolism and should be considered as regulators of fuel selection. Fuel selection and utilization could play a critical role in healthy aging. Characterization of metabolic events in ShcKO mice would help to elucidate how metabolism is influenced by these proteins.

## Highlights

• Decreased glycolysis and increased gluconeogenesis in ShcKO mice.
• Changes in metabolite levels reflect changes in enzyme activities.
• Protein levels of key enzymes remained unchanged.
• Shc proteins play a role in metabolic shift and act as regulators of fuel selection.

## Graphical abstract

**Figure:**
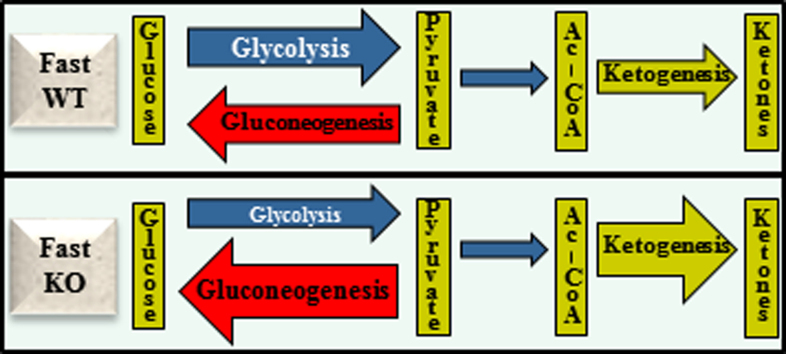

## 1 Introduction

Three adaptor proteins, p66^Shc^, p46^Shc^ and p52^Shc^, are encoded by the Shc locus in mammals and are involved in signal transmission from growth factor receptors [1]. The three isoforms are the result of alternative splicing or translation initiation of the same RNA [1], [2]. Additionally, an independent p66Shc promotor has also been described [3]. Previous reports have implicated Shc proteins in insulin/IGF-1 and receptor tyrosine kinase signaling [1], [4], suggesting that these proteins may play a role in energy metabolism. Initial studies investigating the influence of Shc proteins on whole animal energy metabolism focused on the p66Shc isoform using the p66Shc KO mouse [5], [6]. However, while these mice lack p66Shc, it has been recently shown that they also have decreased levels of p52Shc in all tissues and decreased levels of p46Shc in liver, skeletal muscle and heart [7]. Thus, these mice (referred to as ShcKO hereafter) provide a model of overall decreases in Shc proteins in multiple tissues. The first evidence that Shc proteins may have an important influence on whole animal energy metabolism came from a study showing that ShcKO mice were resistant to weight gain on a high fat diet [5]. Weight gain was also decreased in leptin deficient ShcKO Ob/Ob mice compared to wild-type Ob/Ob animals [6]. Insulin sensitivity and glucose tolerance were also increased in ShcKO mice [6], [7], providing further evidence that Shc proteins modulate whole animal energy metabolism. However, relatively little is known about the influence of Shc proteins on major cellular metabolic pathways. It has recently been shown that changes in enzyme activities and regulatory metabolite levels in ShcKO mice indicate a decreased capacity for glycolysis in skeletal muscle from these animals compared to wild-type controls under both fed and fasting conditions [8]. The activities of fatty acid β-oxidation enzymes in liver and skeletal muscle were also increased in ShcKO compared to wild-type mice in the fasted state, and the activities of ketogenic enzymes were increased in liver from ShcKO versus wild-type mice [9]. Moreover, we have reported recently that the β-oxidation enzyme 3-ketoacyl-CoA thiolase was directly influenced by p46Shc through binding that resulted in decreased activity and that lower levels of p46Shc resulted in increased activity of the enzyme [10]. The results of our current studies are consistent with the idea that Shc proteins may play a role in regulating substrate oxidation.

Liver plays a central role in glucose metabolism, and serves as a major site for glycolysis under fed conditions and functions as the primary site for gluconeogenesis under fasting conditions [11]. In glycolysis, three enzymes, glucokinase, phosphofructokinase-1 and pyruvate kinase, play key roles in its regulation [12], while four enzymes, namely, pyruvate carboxylase, phosphoenolpyruvate carboxykinase, fructose-1,6-bisphosphatase and glucose 6-phosphatase, are involved in the regulation of gluconeogenesis [13]. Additionally, PDH is another key regulatory point by virtue of its position, linking glycolysis to Krebs cycle. While it has recently been shown that the glycolytic capacity was decreased in skeletal muscle from ShcKO mice [8], little is known about the influence of Shc proteins on glycolysis or gluconeogenesis in liver.

The aim of the current study was to establish the role of Shc proteins in the regulation of glycolytic and gluconeogenic enzyme activities in the livers of WT and ShcKO mice under both fed and fasted states. The impact of Shc proteins on various metabolites and other regulatory parameters that exert influence on these pathways was also investigated.

## 2 Materials and methods

### 2.1 Materials and antibodies

General laboratory chemicals and reagents were purchased from Sigma-Aldrich (St. Louis, MO), except bovine serum albumin (MP Biochemicals, Santa Ana, CA), Bio-Rad protein assay dye (BioRad, Hercules, CA), *p*-(*p*-aminophenylazo)-benzene sulphonic acid (Alfa Aesar, Ward Hill, MA) and NAD, NADH and ATP (Roche Diagnostics, Indianapolis, IN). Auxiliary enzymes used in the measurement of glycolytic, gluconeogenic and transamination enzyme activities, as well as in metabolite determinations were from Sigma-Aldrich (St. Louis, MO) or Roche Diagnostics (Indianapolis, IN). Primary antibodies used in this work were rabbit anti-GK antibody (Life Span Biosciences, Atlanta, GA), goat anti-PFK1 (GenWay Biotech, San Diego, CA), mouse anti-AMPK, rabbit anti-pAMPK, rabbit anti-FOXO1 and rabbit anti-Shc (Cell Signaling, Danvers, MA), mouse anti-tubulin (Sigma Aldrich, St Louis, MO), mouse anti-PDH (Mito Sciences, Eugene, OR), goat anti-GKRP, rabbit anti-PEPCK, rabbit anti-FBPase and rabbit anti-pPDH (Novus Biologicals, Littleton, CO), and rabbit anti-PDK4 antibody (kindly provided by Dr. Pengfei Wu, Department of Medicine, Indiana University School of Medicine, Indianapolis, IN).

### 2.2 Animals

The ShcKO mice were provided by Dr. Pier Giuseppe Pelicci (Department of Experimental Oncology, European Institute of Oncology, Milan, Italy), and used for the establishment of a breeding colony at UC Davis, as previously described [8]. Male wild-type and ShcKO mice used in all experiments were housed in a controlled animal facility at 22–24 °C and 40–60% humidity, with a 12 h light: dark cycle. All mice had free access to food (LM485, Teklad, Madison, WI) and water. At 3 months of age, the mice were randomly divided into two groups: fed and fasted. For the fasted group, mice were deprived of food for 16 h and then sacrificed. Mice in the fed group were deprived of food for 16 h followed by 3 h of feeding then sacrificed. All animal care and use protocols were approved by the UC Davis Institutional Animal Care and Use Committee (IACUC) and are in accordance with the National Institutes of Health (NIH) guidelines for the Care and Use of Laboratory Animals.

### 2.3 Tissue harvesting and preparation

Mice were sacrificed by cervical dislocation between 9–10am and weighed. Livers were rapidly removed, weighed immediately and frozen in liquid nitrogen. Frozen livers were then powdered in a porcelain mortar and pestle maintained under liquid nitrogen. All tissue powders were stored under liquid nitrogen.

### 2.4 Gluconeogenic and glycolytic enzyme assays

The activities of gluconeogenic enzymes pyruvate carboxylase (PC, EC 6.4.1.1), phosphoenolpyruvate carboxykinase (PEPCK, EC 4.1.1.32), fructose-1,6-bisphosphatase (FBPase, EC 3.1.3.11) and glucose-6-phosphatase (G6Pase, EC 3.1.3.9) were measured in liver, as previously described [14]. The activities of the glycolytic enzymes glucokinase (GK, EC 2.7.1.1), phosphofructokinase-1 (PFK1, EC 2.7.1.11) and pyruvate kinase (PK, EC 2.7.1.40) were also measured in liver as previously described [15]. All assays were performed using a Perkin Elmer Lambda 25 UV/Vis spectrophotometer equipped with a Peltier heating control system and 9 cell changer (Perkin Elmer, Shelton, CT). Reactions were followed at 340 nm (ε=6.22 mM^−1^.cm^−1^) for GK, PFK1, PK, FBPase and PEPCK, 412 nm (ε=13.6 mM^−1^.cm^−1^) for PC and 510 nm (ε=6.66 mM^−1^.cm^−1^) for G6Pase. All enzyme activities were expressed as μmol/min/mg protein, and presented as mean±SEM determined from at least six animals.

### 2.5 Pyruvate dehydrogenase complex assay

The activity of pyruvate dehydrogenase complex (PDH, EC 1.2.4.1+EC 2.3.1.12+EC 1.8.1.4) was assayed spectrophotometrically at 460 nm (ε=7.11 mM^−1^cm^−1^), by coupling to the activity of arylamine acetyltransferase (AAT, EC 2.3.1.5) and the dye *p*-(*p*-aminophenylazo)-benzene sulphonic acid (AABS), as previously described [8]. Activities were expressed as μmol/min/mg protein and presented as mean±SEM determined from at least six animals.

### 2.6 Transamination enzyme assays

Alanine transaminase (ALA, EC 2.6.1.2), aspartate transaminase (ASP, EC 2.6.1.1), tryptophan transaminase (TRT, EC 2.6.1.27) and tyrosine transaminase (TYT, EC 2.6.1.5) were assayed as previously described [14]. The assays were performed spectrophotometrically, and the reactions were followed at 340 nm (ε=6.22 mM^−1^.cm^−1^) for ALA and ASP, 328 nm (ε=14 mM^−1^.cm^−1^) for TRT and 310 nm (ε=9.85 mM^−1^.cm^−1^) for TYT. Activities were expressed as μmol/min/mg protein and presented as mean±SEM determined from at least six animals.

### 2.7 Glucose and glycogen levels

Liver glucose and glycogen levels were determined based on measuring both free glucose levels and glucose resulting from glycogen hydrolysis as previously described [8]. Liver powders were homogenized in 6% (w/v) perchloric acid and a small portion was taken for glycogen hydrolysis and the remainder used for glucose determination, as described in the indicated method. Total and free glucose levels were determined at 340 nm (ε=6.22 mM^−1^cm^−1^). Subtraction of free glucose from the total glucose gave glucose levels resulting from glycogen hydrolysis, hence glycogen levels. Concentrations were presented as mean±SEM determined from at least six animals and values expressed as μmol/g wet weight.

### 2.8 Metabolite levels

Frozen liver powders were homogenized in 6% (w/v) perchloric acid, except for F2,6BP which was homogenized in 0.05 M NaOH, according to the indicated methods. The metabolites G6P, F6P, F1,6BP, F2,6BP, PEP, Pyr, Lac and ketone bodies were measured as previously described [15]. Acetyl-Co and CoA [14], fructose and fructose-1-phosphate [16], as well as malate and α-ketoglutarate [17] were also measured as indicated. All values were expressed as μmol/g wet weight, except for fructose-2,6-bisphospahte which was expressed as nmol/g wet weight. All data were presented as mean±SEM from at least six animals.

### 2.9 Electrophoresis and western blotting

Total proteins were prepared using lysis buffer (Cell Signaling Technologies, Danvers, MA) and resolved by SDS–PAGE electrophoresis. Proteins were transferred to nitrocellulose membrane using the iBlot system (Life Technologies, Calrsbad, CA) and blocked with Odyssey Blocking Buffer (Li-Cor Biosciences, Lincoln, NE). The membranes were probed with the following primary antibodies, using the indicated dilution: rabbit anti-GK (1/500), goat anti-PFK (1/1000), anti-PEPCK (1/1000), anti FBPase (1/500), rabbit anti-pAMPK (1/500), mouse anti-AMPK (1/1000), rabbit anti-FOXO1 (1/500), mouse anti-tubulin (1/5000), rabbit anti-PDK4 (1/500), mouse anti-PDH (1/1000), rabbit anti-pPDH (1/500), and goat anti-GKRP (1/2000). This was followed by treatment with infrared IR-dye 700CW and/or 800CW-labeled secondary antibodies (Li-Cor Biosciences, Lincoln, NE). The blots were then scanned and protein levels quantified using Li-Cor Odyssey infrared imaging instrument and Odyssey 2.1 software.

### 2.10 Protein assays

Protein concentrations were determined using the Bio-Rad protein assay kit (Bio-Rad Laboratories, Hercules, CA), with BSA as the standard.

### 2.11 Statistical analysis

Differences between genotypes and dietary regimens were evaluated with a two-way ANOVA with genotype and dietary regimen as main effects and including the interaction between these factors. All analyses were conducted in R version 3.02. Where the overall ANOVA was significant, we identified genotypes and dietary regimens that differed significantly using Tukey's multiple comparison procedure and maintained the family-wise error rate at 0.05.

## 3 Results

### 3.1 Activities of glycolytic enzymes and PDH complex

**Fig. 1:**
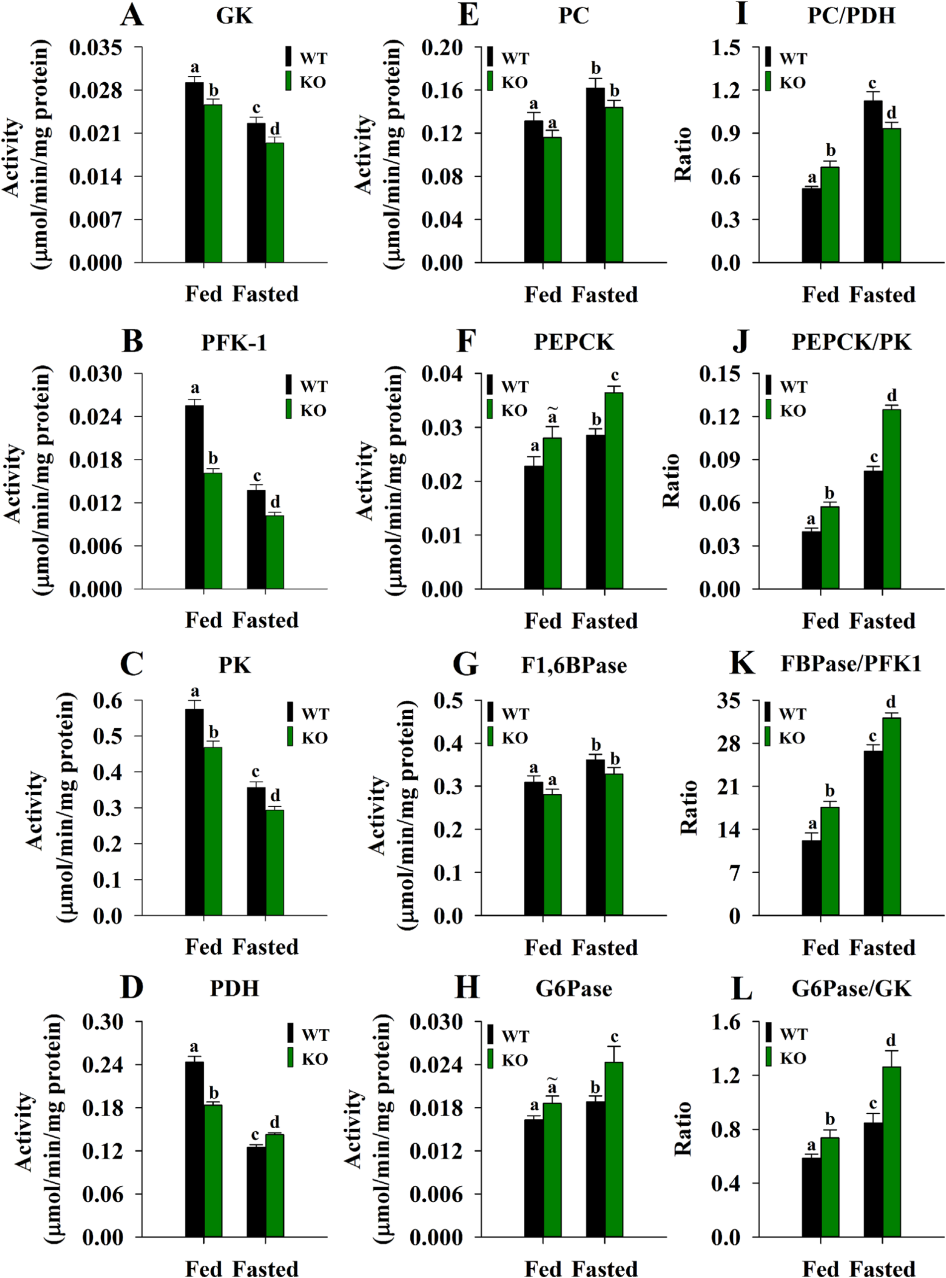
Glycolytic and gluconeogenic enzyme activities and their ratios in livers from ShcKO and WT mice under fed or fasted states. All values are mean±SEM (n =6) and presented as μmol/min/mg protein. The following comparisons were made: within a genotype, fed versus fasted; across genotypes, fed versus fed and fasted versus fasted. Bars that do not share a common symbol differ significantly (*P*<0.05). The symbol (~) is used to indicate a trend towards an increase or a decrease (*P*<0.10) when the difference is not significant.

The enzymes GK, PFK1 and PK were assayed from livers of WT and ShcKO mice (Fig. 1A–C, respectively). All three enzymes showed lower activities (*P*<0.05) in the ShcKO mice under both fed and fasting states when compared to WT mice. Moreover, under the fasting state, activities of all three enzymes in both WT and ShcKO mice were lower (*P*<0.05) than activities under the fed state. PDH complex activity (Fig. 1D) was lower (*P*<0.05) in the ShcKO versus WT mice under the fed state. Fasting resulted in decreased activities (*P*<0.05) in both WT and ShcKO mice, however, the fasted ShcKO mice displayed higher activities (*P*<0.05) than the fasted WT mice.

### 3.2 Activities of gluconeogenic enzymes

The activities of the four enzymes were measured from livers of WT and ShcKO mice (Fig. 1). PC and FBPase activities (Fig. 1E and G) were not different between WT and ShcKO mice in the fed or fasted states. PEPCK activity (Fig. 1F) in the ShcKO mice showed a trend towards an increase (*P*=0.089) in the fed state compared to WT, while in the fasted state it was higher (*P*<0.05) than the WT. The activity of G6Pase (Fig. 1H) was similar to the PEPCK activity patterns, with ShcKO mice showing a trend towards an increase (*P*=0.076) in the fed state and a higher activity (*P*<0.05) in the fasted state when compared to their WT counterparts. For all four enzymes, under fasting conditions, activities in both WT and ShcKO mice were higher (*P*<0.05) than fed mice.

### 3.3 Ratios of gluconeogenic to glycolytic enzymes

The ratios of gluconeogenic to glycolytic enzyme activities were determined as indicators of increased gluconeogenesis. The ratios PEPCK/PK (Fig. 1J), FBPase/PFK1 (Fig. 1K) and G6Pase/GK (Fig. 1L) were higher (*P*<0.05) in the fasted state indicating increased gluconeogenesis, and were higher (*P*<0.05) in the ShcKO versus WT mice under both fed and fasting states. However, the ratio of PC/PDH (Fig. 1I) was higher (*P*<0.05) in WT and ShcKO mice in the fasted versus fed state while in the fasted state this ratio was lower (*P*<0.05) in the ShcKO versus WT consistent with higher PDH activity in the fasted ShcKO animals.

### 3.4 Activities of transaminase enzymes

**Fig. 2:**
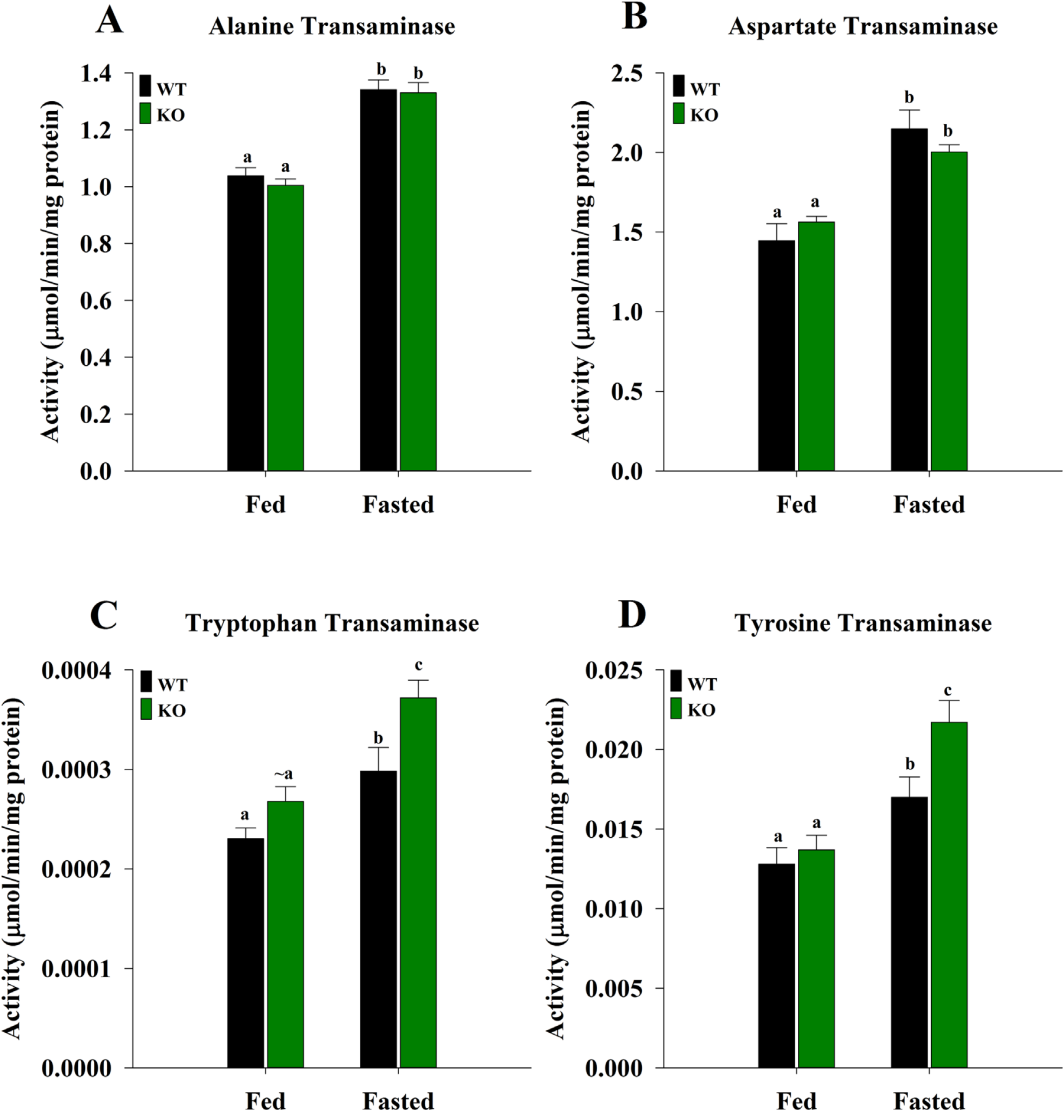
The activities of transamination enzymes in the livers from ShcKO and WT mice under fed or fasted states. All values are mean±SEM (n=6) and presented as μmol/min/mg protein. The following comparisons were made: within a genotype, fed versus fasted; across genotypes, fed versus fed and fasted versus fasted. Bars that do not share a common symbol differ significantly (*P*<0.05). The symbol (~) is used to indicate a trend towards an increase or a decrease (*P*<0.10) when the difference is not significant.

Several transaminase enzyme activities were also determined (Fig. 2). All transaminase enzyme activities were increased (*P*<0.05) in the fasted versus fed state for both WT and ShcKO mice. There were no differences in ALT and AST activities (Fig. 2A and B) between WT and ShcKO mice in either the fed or fasted states. In contrast, TRT activity (Fig. 2C) in ShcKO mice showed a trend towards an increase (*P*=0.07) in the fed state and was increased (*P*<0.05) in the fasted state when compared to WT mice. TYT activity (Fig. 2D) was also increased (*P*<0.05) in fasted ShcKO compared to WT mice, while there were no differences between the genotypes in the fed state.

### 3.5 Levels of glucose and glycogen in liver

**Fig. 3:**
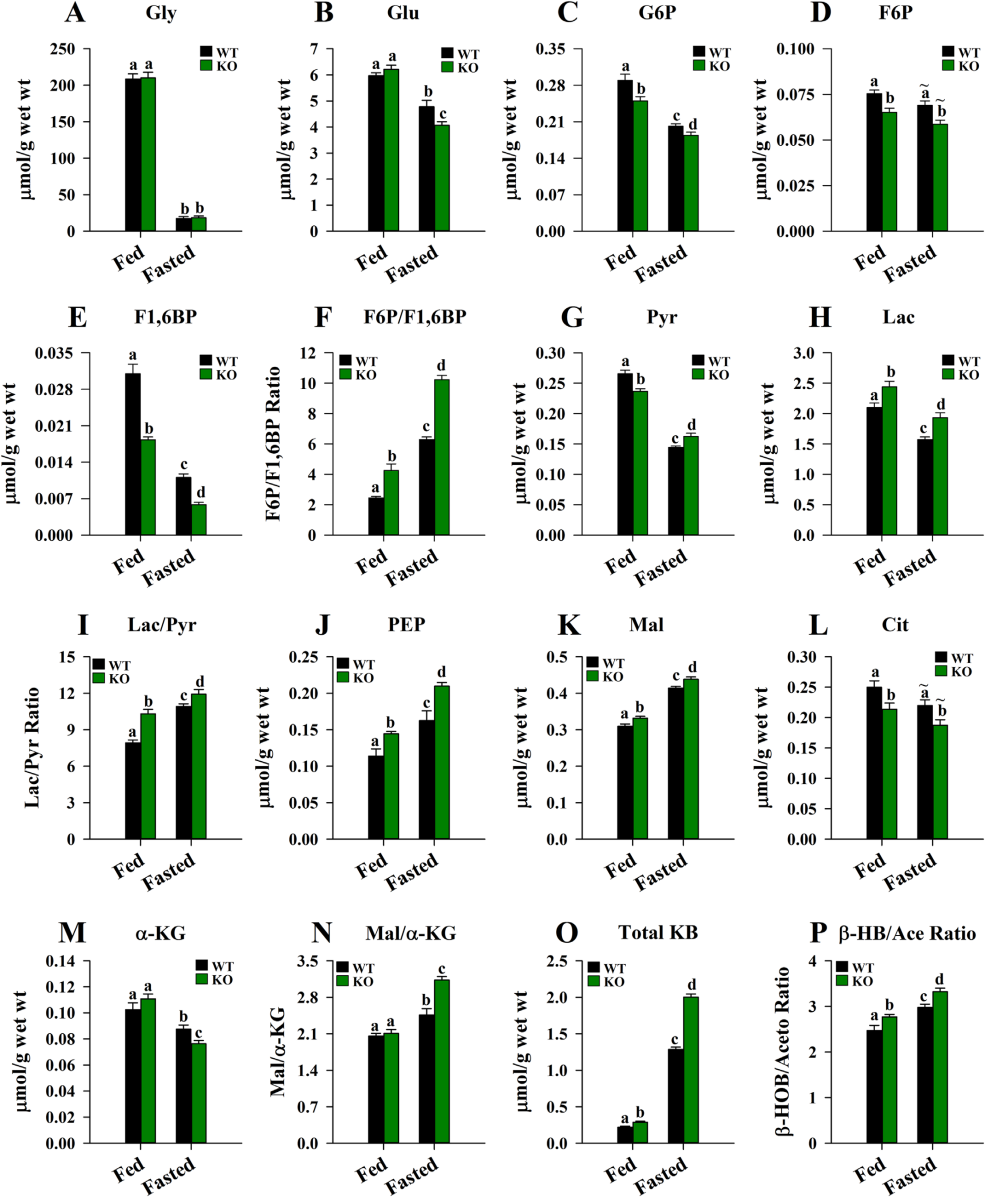
Levels of glycolytic metabolites, total ketone bodies and other metabolites in the livers from ShcKO and WT mice under fed or fasted states. All values are mean±SEM (n=6) and presented as μmol/g wet weight. The following comparisons were made: within a genotype, fed versus fasted; across genotypes, fed versus fed and fasted versus fasted. Bars that do not share a common symbol differ significantly (*P*<0.05). The symbol (~) is used to indicate a trend towards an increase or a decrease (*P*<0.10) when the difference is not significant.

Hepatic levels of glucose and glycogen were measured in the WT and ShcKO mice (Fig. 3). Glycogen (Fig. 3A) and glucose (Fig. 3B) levels were lower (*P*<0.05) in the fasted versus fed state for both genotypes. Glycogen levels (Fig. 3A) were not different between WT and ShcKO mice in the fed or fasted state. No differences in glucose levels (Fig. 3B) were observed between WT and ShcKO mice in the fed state, while in the fasted state the ShcKO mice had lower (*P*<0.05) glucose levels compared to WT.

### 3.6 Liver metabolite levels

The levels of several metabolites of the glycolytic/gluconeogenic pathways from liver were measured (Fig. 3). When comparing ShcKO and WT animals, the ShcKO mice displayed lower (*P*<0.05) G6P, F6P and F1,6 BP levels versus WT mice in both fed and fasted states (Fig. 3C, D and 3E, respectively). The ratio of F6P/F1,6BP (Fig. 3F), an indicator of PFK1 inhibition, was higher (*P*<0.05) in the ShcKO versus WT mice in both fed and fasted states. Pyruvate levels (Fig. 3G) in the ShcKO versus WT mice were lower (*P*<0.05) in the fed state but higher (*P*<0.05) in the fasted state, while lactate levels (Fig. 3H) were higher (*P*<0.05) in ShcKO versus WT mice in both fed and fasted states. The Lactate/Pyruvate ratio (Fig. 3I) was higher (*P*<0.05) in the ShcKO versus WT mice under both fed and fasted states. PEP (Fig. 3J) and malate (Fig. 3K) showed similar patterns and were the only metabolites that increased (*P*<0.05) with fasting in both genotypes (Fig. 3J and L), and were higher (*P*<0.05) in ShcKO versus WT mice. Citrate levels (Fig. 3L) were lower (*P*<0.05) in the ShcKO versus WT mice under both fed and fasted states, but showed a trend toward a decrease with fasting in both the WT (*P*<0.065) and ShcKO (*P*<0.089) mice. α-Ketoglutarate levels (Fig. 3M) were similar for WT and ShcKO mice in the fed state but were lower (*P*<0.05) in the fasted ShcKO, and the ratio of malate/α-ketoglutarate (Fig. 3N) was higher (*P*<0.05) in ShcKO animals in the fasted state.

### 3.7 Levels of ketone bodies

Total ketone body concentrations in the livers (Fig. 3O) were higher (*P*<0.05) in ShcKO versus WT mice in both fed and fasted states. Fasting increased (*P*<0.05) the levels even further for both ShcKO and WT mice. The β-hydroxybutyrate to acetoacetate ratio (Fig. 3P), an indicator of mitochondrial redox state, was also higher (*P*<0.05) in the ShcKO versus WT mice, in both fed and fasted states. Fasting increased (*P*<0.05) the ratios in both genotypes.

### 3.8 Levels of acetyl-CoA and CoA in liver

**Fig. 4:**
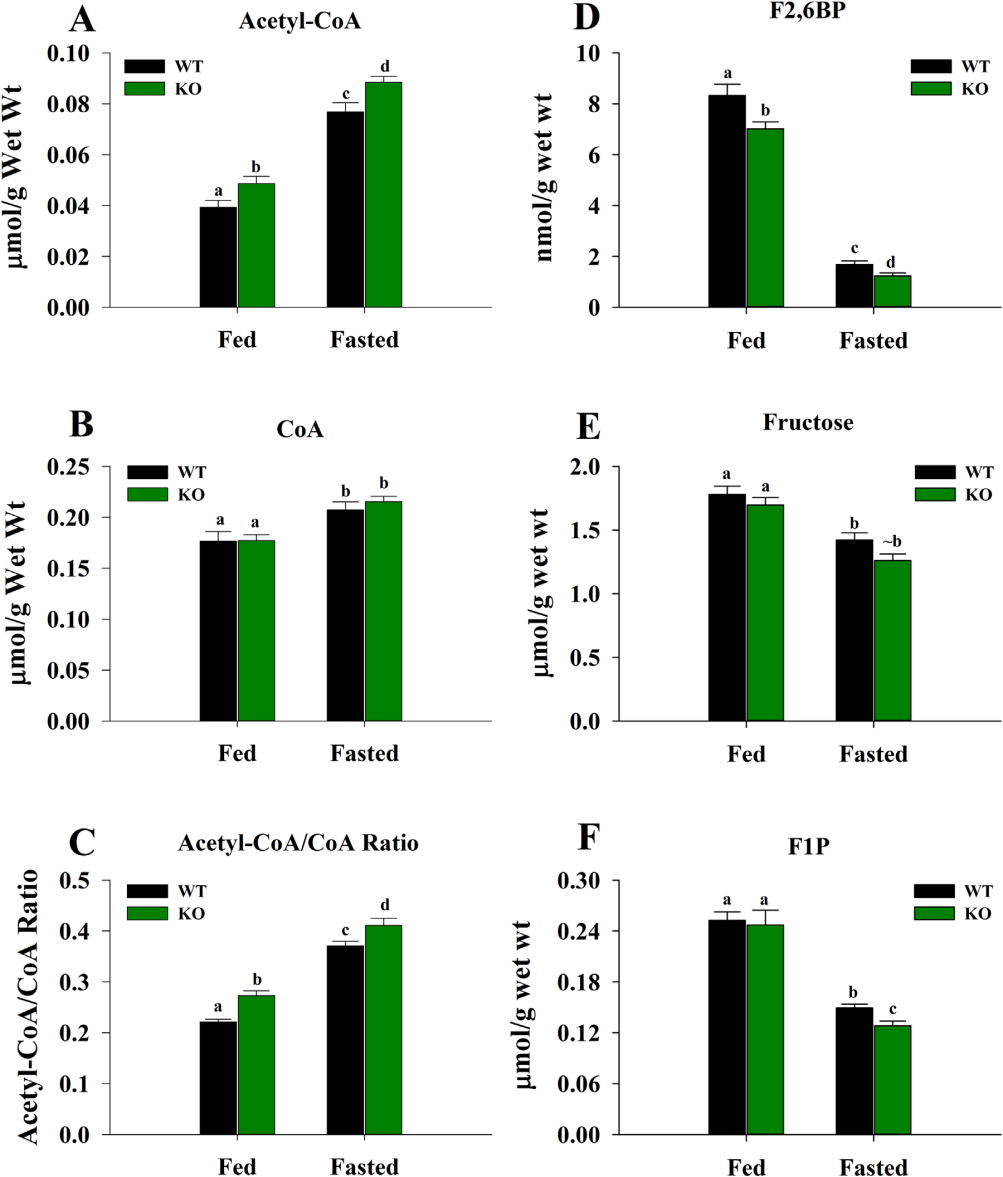
Levels of acetyl-CoA, CoA, F2,6BP, fructose and F1P in the livers from ShcKO and WT mice under fed or fasted states. All values are mean±SEM (n=6) and presented as μmol/g wet weight, except for F2,6BP which was presented as nmol/g wet weight. The following comparisons were made: within a genotype, fed versus fasted; across genotypes, fed versus fed and fasted versus fasted. Bars that do not share a common symbol differ significantly (*P*<0.05). Data presented as mean±SEM (n=6). The symbol (~) is used to indicate a trend towards an increase or a decrease (*P*<0.10) when the difference is not significant.

Acetyl-CoA levels (Fig. 4A) were higher (*P*<0.05) in the ShcKO versus WT mice under both fed and fasted states. Fasting increased acetyl-CoA levels, with both WT and ShcKO mice displaying higher (*P*<0.05) levels versus their fed counterparts. No differences in the CoA levels (Fig. 4B) were observed between WT and ShcKO mice, under both fed and fasted states, but the levels were higher (*P*<0.05) under fasted versus fed state in both WT and ShcKO mice. The ratio of acetyl-CoA/CoA (Fig. 4C) showed similar patterns to that of the acetyl-CoA, with higher ratios in fed and fasted ShcKO versus WT mice.

### 3.9 Levels of fructose-2,6-bisphosphate, fructose and fructose-1-phosphate in liver

Liver F2,6BP levels (Fig. 4D) were lower (*P*<0.05) in the ShcKO versus WT mice under both fed and fasted states. Moreover, in the fasted state, both WT and ShcKO mice showed decreased levels (*P*<0.05) compared to the fed mice. Fructose levels (Fig. 4E) showed no differences between WT and ShcKO mice under the fed state, but under the fasted state, a trend towards a decrease (*P*=0.06) in the ShcKO versus WT mice was observed. Fasting decreased (*P*<0.05) fructose levels in both WT and ShcKO mice when compared to the corresponding fed mice. In the case of F1P (Fig. 4F), no differences in the levels were observed between WT and ShcKO mice in the fed state, however, in the fasted state, the levels in ShcKO were lower (*P*<0.05) than the WT.

### 3.10 Protein Levels of the glycolytic and gluconeogenic enzymes, and GKRP in liver

**Fig. 5:**
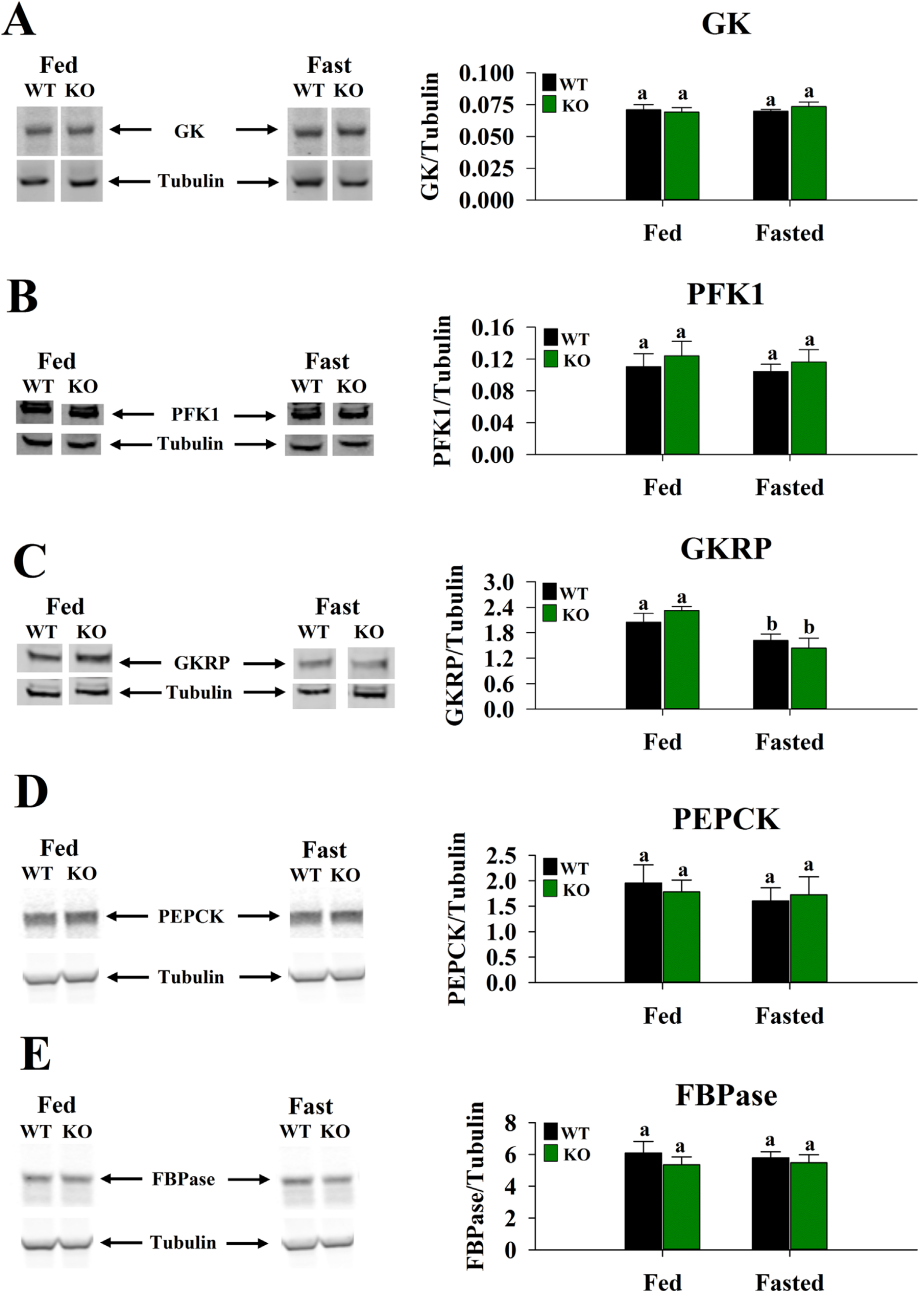
Protein levels of GK, PFK1, GKRP, PEPCK and FBPase in the livers from ShcKO and WT mice under fed or fasted states. Western blotting was performed as described. All values are mean±SEM (n=6). The following comparisons were made: within a genotype, fed versus fasted; across genotypes, fed versus fed and fasted versus fasted. Bars that do not share a common symbol differ significantly (*P*<0.05).

The protein levels were determined by western blotting (Fig. 5). GK, PFK-1, GKRP, PEPCK and FBPase did not show any differences between WT and ShcKO mice in the fed or fasted states (Fig. 5). However, with GKRP (Fig. 5C), lower levels (*P*<0.05) were observed in the fasted versus fed states in both the WT and ShcKO mice.

### 3.11 Protein Levels of PDH, p-PDH and PDK4

**Fig. 6:**
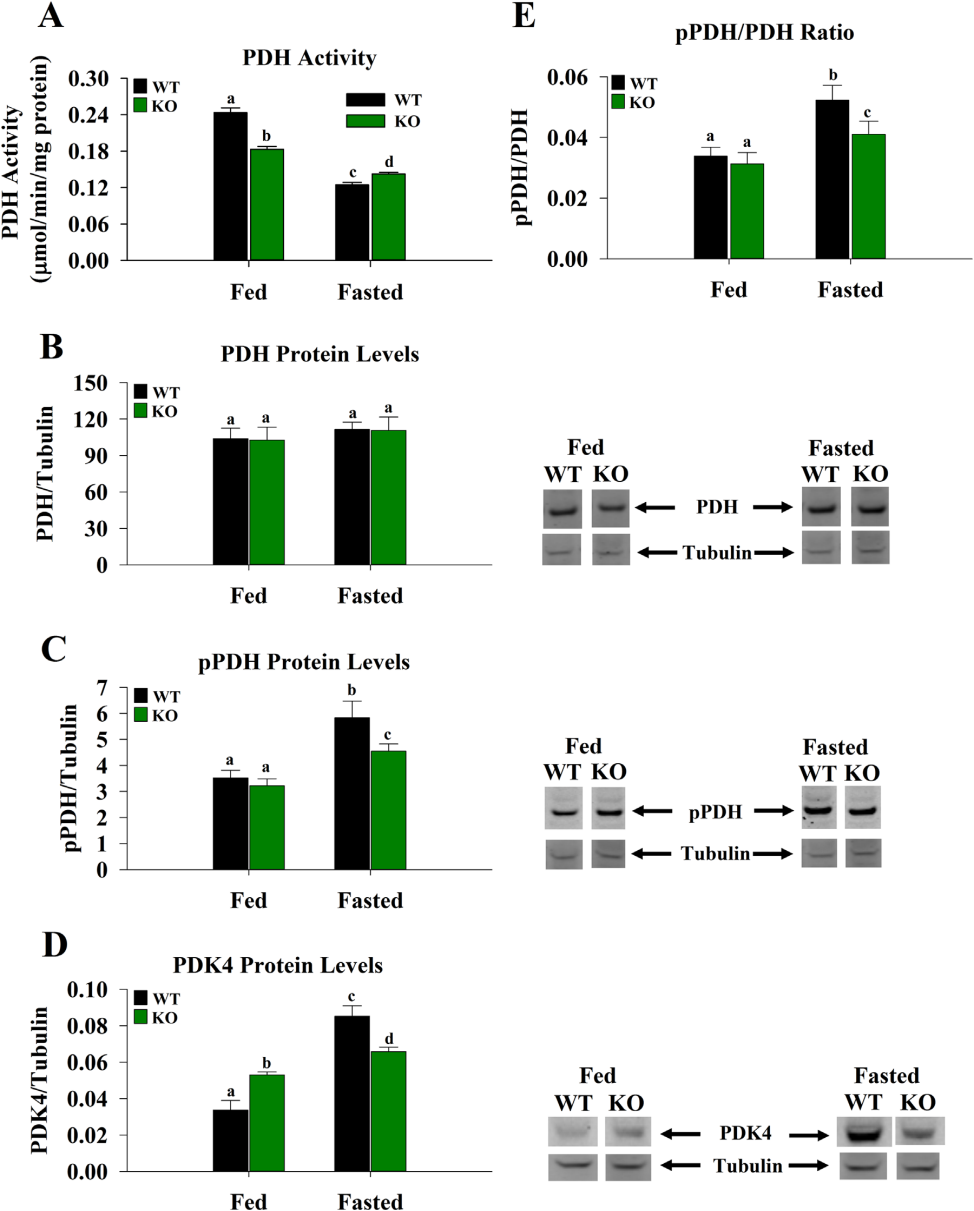
Protein levels of PDH, pPDH and PDK4 in the livers from ShcKO and WT mice under fed or fasted states. Western blotting was performed as described. The activity of PDH (A) is presented here for comparison. All values are means±SEM. The following comparisons were made: within a genotype, fed versus fasted; across genotypes, fed versus fed and fasted versus fasted. Bars that do not share a common symbol differ significantly (*P*<0.05).

Western blotting of liver samples indicated that there were no differences in the levels of PDH between WT and ShcKO in the fed or fasted states (Fig. 6B). The levels of liver pPDH (Fig. 6C) were also not different between WT and ShcKO mice in the fed state, however, the levels were increased (*P*<0.05) in the fasted state, with the WT levels being higher (*P*<0.05) than the ShcKO. In the case of PDK4 (Fig. 6D), higher levels (*P*<0.05) were observed in the ShcKO versus WT in the fed state. Fasting increased the levels of PDK4 (*P*<0.05) in WT and ShcKO mice, but the fasted ShcKO mice showed levels that were lower (*P*<0.05) than the fasted WT mice. This pattern of PDK4 corresponds with the observed PDH activities (Fig. 6A). The results also showed that the pPDH/PDH ratio (Fig. 6E) was unchanged between the fed WT and ShcKO mice but increased with fasting, with the ratio being higher (*P*<0.01) in the WT versus ShcKO mice.

### 3.12 Protein Levels of AMPK, pAMPK and FOXO1

**Fig. 7:**
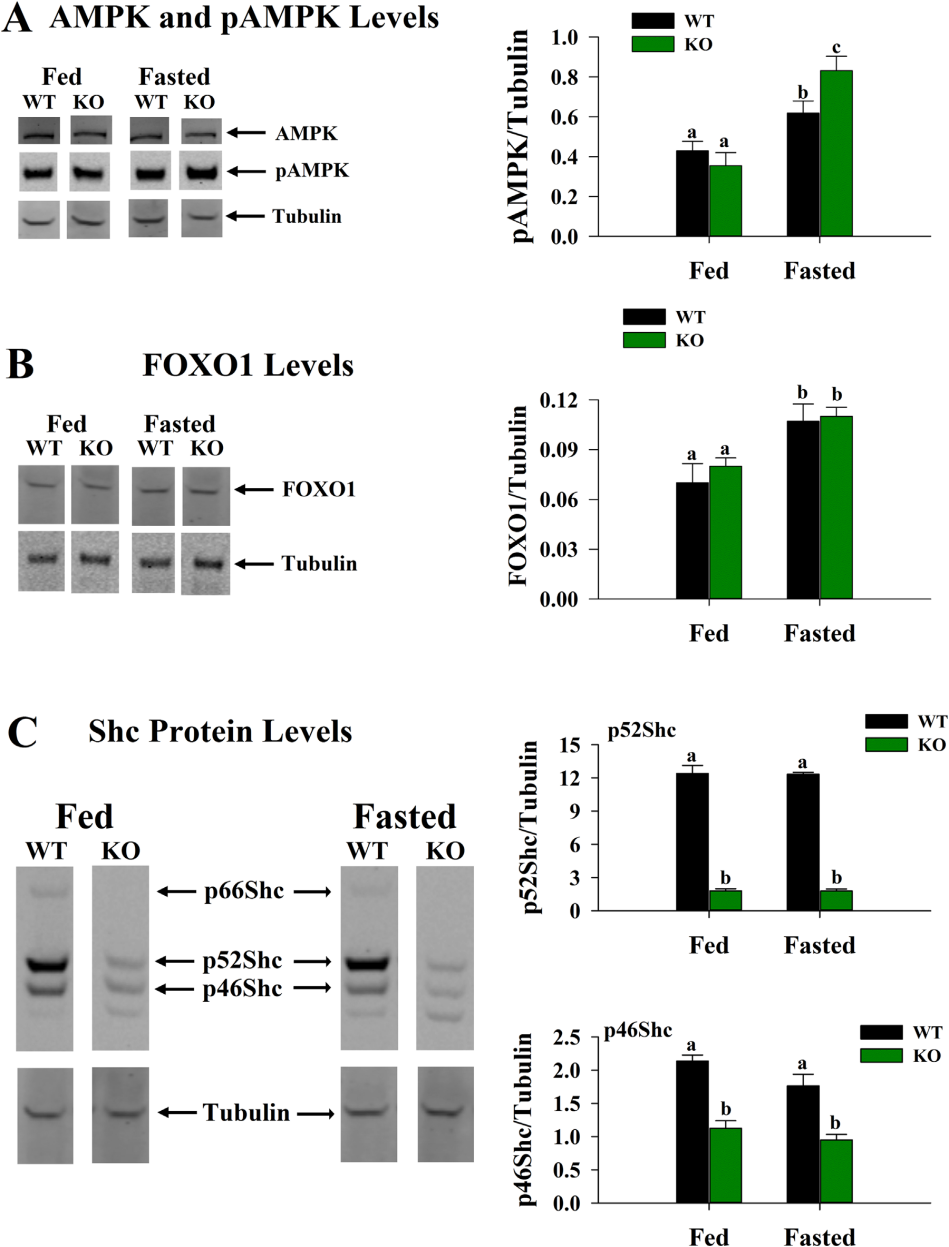
Protein levels of pAMPK, FOXO1 and Shc proteins in the livers from ShcKO and WT mice under fed or fasted states. Western blotting was performed as described. All values are means±SEM. The following comparisons were made: within a genotype, fed versus fasted; across genotypes, fed versus fed and fasted versus fasted. Bars that do not share a common symbol differ significantly (*P*<0.05).

The protein levels of AMPK, pAMPK and FOXO1 were determined by western blotting (Fig. 7). Levels of AMPK remained unchanged in both genotypes under fed and fasted states (Fig. 7A), but fasting increased (*P*<0.05) pAMPK levels (Fig. 7A) in both WT and ShcKO mice when compared to fed mice, with ShcKO mice having higher levels (*P*<0.05) than the WT. However, there were no differences between the fed WT and ShcKO mice. Fasting increased (*P*<0.05) FOXO1 levels (Fig. 7B) in both the ShcKO and WT mice compared to the fed, however, there were no differences between the two genotypes in the fed or fasted states.

### 3.13 Levels of Shc proteins in liver

Liver p66Shc, p52Shc and p46Shc proteins were determined by western blotting (Fig. 7C). The levels of p66Shc were undetectable in the ShcKO mice in both the fed and fasted states. However, the levels of p52Shc and p46Shc were also lower (*P*<0.05) in the ShcKO versus WT mice in both the fed and fasted states.

## 4 Discussion

**Fig. 8:**
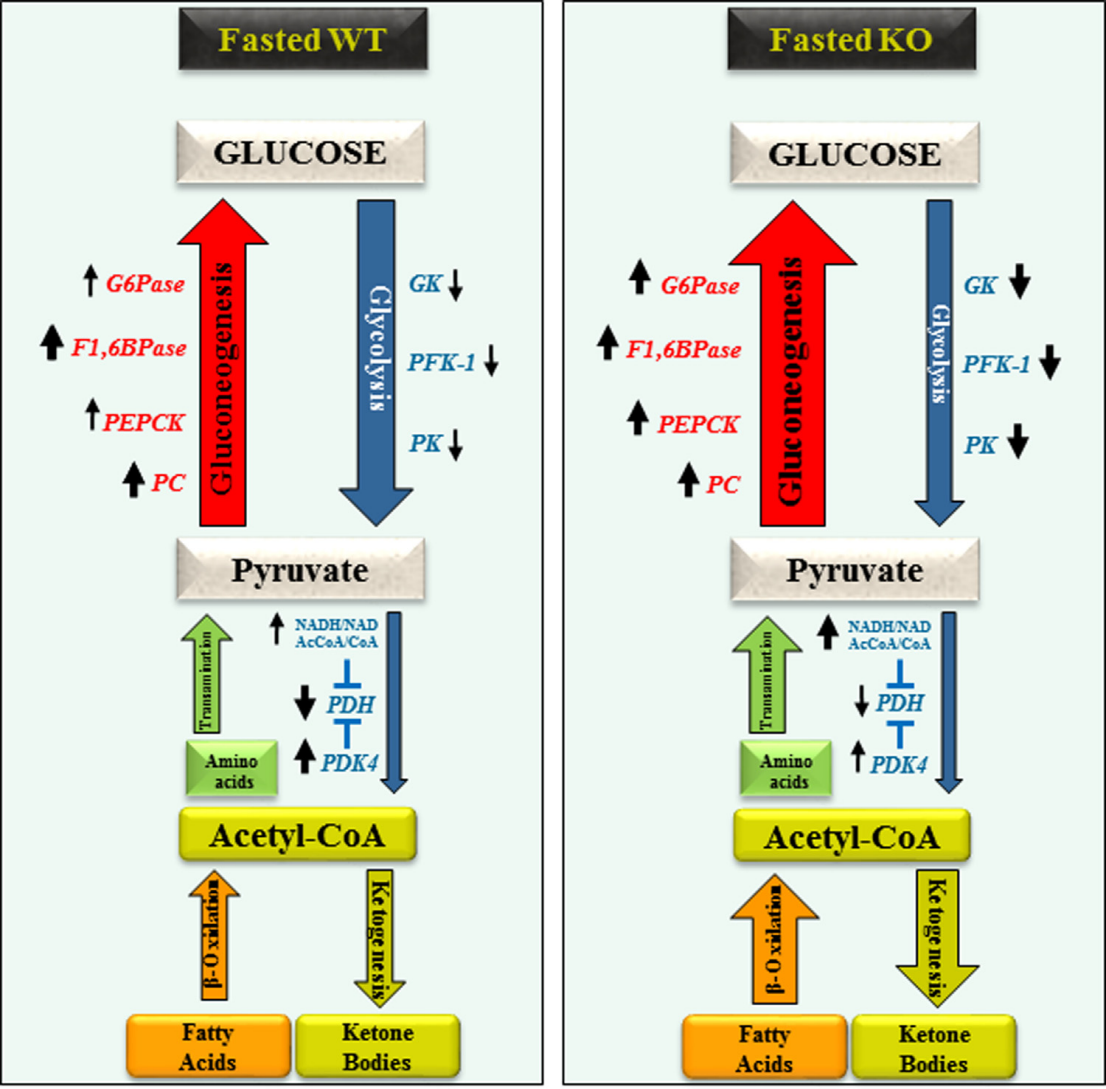
The integration of glycolytic, gluconeogenic, PDH, transamination and ketogenesis pathways in the livers of fasted WT (left panel) and fasted KO (right panel) mice. Up or down black arrows indicate increased or decreased activities, respectively, of a given enzyme/metabolite ratio when compared with their corresponding fed state. Thickness of the up or down black arrows indicates the magnitude of the increased or decreased activity of a given enzyme/metabolite ratio in the fasted state when comparison is made between the fasted WT and ShcKO mice. The thickness of the pathway arrows indicates the increased or decreased capacity of the specific pathway under the specific nutritional state.

The results of the present study show a decrease in capacity for glycolysis and an increase in capacity for gluconeogenesis in ShcKO versus WT mice based on the activity of key regulatory enzymes in these pathways. We have previously reported in skeletal muscle that capacity for glycolysis was decreased in ShcKO mice [8], while β-oxidation enzyme activities were increased [9]. These results are consistent with the idea that Shc proteins play a role in fuel selection. The present study shows that Shc-related shifts in the activities of enzymes in major metabolic pathways are not limited to skeletal muscle but also occur in liver. In liver, when glucose availability becomes limiting, the activity of the glycolytic pathway is suppressed to prevent the oxidation of the available glucose and preserve it for other tissues, such as the brain. This leads to the activation of the gluconeogenic pathway, which is accompanied by increased transamination so that amino acids could be utilized as sources of energy. Decreased Shc levels alter enzyme activities in a manner which creates an environment which would promote the transition from glycolysis to gluconeogenesis when supply of glucose is restricted. The integration of the glycolytic, gluconeogenic, PDH, transamination and ketogenesis pathways is presented in Fig. 8.

The mechanisms through which Shc proteins influence the activities of glycolytic and gluconeogenic enzymes are not known. However, similar to skeletal muscle [8], Shc-related changes in enzyme activity do not appear to be due to alterations in gene expression since there were no differences in the protein levels of glycolytic or gluconeogenic enzymes between ShcKO and WT mice. We have recently reported that the activity of thiolase was decreased through direct binding with p46Shc [10] and it is possible that the activities of other enzymes could be altered through binding with Shc proteins. Additional studies are needed to determine if Shc proteins bind and directly influence the activities of any enzymes in the glycolysis or gluconeogenesis pathways. It is also possible that the Shc-related changes in enzyme activity are due to post-translational modifications, since glycolytic and gluconeogenic enzyme are regulated by phosphorylation [18], [19] and acetylation [20], [21]. In the present study, markers of redox state consistently indicated that the cytosolic (Lac/Pyr) and mitochondrial (β-HB/Ace) compartments were more reduced in the ShcKO compared to wild-type mice. Future studies are needed to determine if the increased NADH/NAD ratio expected under these conditions would alter sirtuin activity and enzyme acetylation in a manner which could contribute to the enzyme activity changes observed in the ShcKO mice.

To determine if the Shc-related changes in the activities of the enzymes at the major control points in glycolysis and gluconeogenesis are consistent with an alteration in metabolism, the levels of several metabolites were measured and enzyme activity ratios were determined. Changes in metabolite levels in the ShcKO compared to WT mice were consistent with a decrease in glycolysis and increase in gluconeogenesis in the ShcKO animals. In particular, the F6P/F1,6BP ratio was significantly increased in ShcKO mice under both fed and fasted conditions indicating decreased activity of PFK1, a major regulator of glycolysis [22]. Also, the higher enzyme activity ratios of PEPCK/PK, FBPase/PFK1 and G6Pase/GK in liver from ShcKO compared to WT mice all provide an indication of increased gluconeogenesis [23]. Thus, metabolite levels and enzyme activity ratios indicate that glycolysis is decreased and gluconeogenesis increased in the ShcKO animals.

Metabolites may also influence metabolism by acting as inhibitors or activators of enzymes. The decreases in GK, PFK1 and PK activities and increases in PEPCK and G6Pase activities in ShcKO liver would likely be amplified in vivo by the changes in regulatory metabolites that occurred in these mice. GK is inhibited when bound to GKRP and F1P removes this inhibition by inducing dissociation of GKRP from GK [24]. The decrease in F1P levels during fasting in ShcKO vs WT liver would thus be ineffective at dissociating GKRP from GK and decrease GK activity in these animals. GK has also been reported to be inhibited by lactate [25] and the increased lactate levels under both fed and fasted states would also be expected to contribute to decreased GK activity in ShcKO livers. GK has also been reported to be inhibited by long-chain fatty acyl-CoAs [26], [27], and we have previously reported that β-oxidation is stimulated in p66ShcKO mice and total ketone body levels are increased in these animals [9] supporting the idea that GK would be inhibited due to fatty acid mobilization. Thus, the metabolite changes which occur with ShcKO are consistent with a decrease in GK activity.

PFK1 is a major regulatory site that controls glycolysis by a complex allosteric regulation [22]. PFK1 is activated by the metabolite F2,6BP which is produced and degraded by the bifunctional enzyme 6-phosphofructo-2-kinase/fructose-2,6-bisphosphatase [28], [29]. The decrease in F2,6BP levels in liver under fed and fasted conditions in ShcKO versus WT mice are consistent with low PFK1 activity in the ShcKO animals. PFK1 is also inhibited by lactate [25], [30], and the increased levels of lactate in the ShcKO livers would be expected to contribute to its inhibition. Taken together the low F2,6BP and high lactate levels in ShcKO compared to WT liver would be expected to create an environment which inhibits PFK1 activity.

Liver PK is regulated allosterically by F1,6BP, the product of PFK1, in a feed-forward activation mechanism [18], [31]. Under the fasted state, with prevailing gluconeogenesis, control of PK activity is critical for preventing the pathway from proceeding towards pyruvate production. Hence, the decline in the level of F1,6BP plays an important role in controlling PK activity and allowing gluconeogenesis to proceed. Higher levels of acetyl-CoA as observed in ShcKO liver are also inhibitory to PK activity and are in agreement with previous reports that the activities of enzymes of β-oxidation and ketone body metabolism are increased in skeletal muscle and liver from ShcKO compared to WT mice [9], [32]. These results are consistent with ShcKO playing a role in shifting metabolism away from glucose catabolism toward fatty acid oxidation and gluconeogenesis.

PDH plays a pivotal role in metabolism by linking the glycolytic pathway to Krebs cycle, and its regulation is controlled not only by phosphorylation/dephosphorylation but also by changes in metabolite levels [33], [34], [35]. The PDH activity pattern in liver was similar to those previously reported in ShcKO and WT skeletal muscle [8] where it was lower in ShcKO versus WT muscle in the fed state while the decrease in PDH activity with fasting was blunted in the ShcKO mice resulting in higher activities in these animals compared to WT in the fasted state. Acetyl-CoA and NADH, products of the reaction, influence the activity of PDH through feedback inhibition. However, these metabolites are also produced by increased β-oxidation [9], and also allosterically activate PDK which in turn phosphorylates and inhibits PDH, as seen in this study. Thus, PDH activity is influenced by both metabolites and phosphorylation through PDK. In the present study, the fed state decrease in PDH activity in the ShcKO versus WT mice was not due to PDK since pPDH levels were not different between genotypes. The decrease in fed state PDH activity in the ShcKO liver was likely due to post-translational modifications other than phosphorylation with metabolite inhibition (acetyl-CoA and NADH) possibly further contributing to changes in enzyme activity in vivo. With fasting, decreases in PDH activity in both genotypes match PDK4 and pPDH levels. Therefore, differences in PDH activity between genotypes during fasting appear to be due primarily to lower PDK4 levels in the ShcKO liver. The blunted decrease in PDH activity with fasting in the ShcKO animals likely reflects the fact that gluconeogenesis is increased in these mice and the decrease in PDH activity needs to be limited to allow for entry of lactate and amino acids into gluconeogenesis.

The activities of gluconeogenic enzymes and metabolite levels provide further support for the idea that gluconeogenesis is increased in liver from ShcKO versus WT mice. PEPCK is the rate limiting enzyme in the gluconeogenic pathway and its activity is increased in fasted ShcKO compared to WT animals. PEPCK is not regulated allosterically or by phosphorylation/dephosphorylation [36]. Nutrients, such as glucose and amino acids, indirectly regulate PEPCK activity by influencing acetylation of the enzyme [20]. In particular, amino acids diminish acetylation and render the protein more stable and resistant to ubiquitin-mediated degradation [21], such as during fasting when gluconeogenesis is needed. Alterations in activity of PEPCK in ShcKO liver do not appear to be due to increases in level of PEPCK protein and additional studies are needed to determine if Shc may influence PEPCK activity through post-transitional modifications. Further support for an increase in gluconeogenesis in the ShcKO mice is provided by the ratios of gluconeogenic to glycolytic enzyme activities. Higher ratios of PEPCK/PK, FBPase/PFK1 and G6Pase/GK provide an indication of increased gluconeogenic activity [23], and each of these ratios were increased in ShcKO compared to WT mice. Shifts in regulatory metabolite levels under fasting conditions were also consistent with increased gluconeogenic enzyme activities in the ShcKO animals. In particular, α-KG has been reported to be an inhibitor of PEPCK [37] and F1P is an inhibitor of G6Pase [38], and the levels of each of these inhibitory metabolites are decreased in liver from fasted ShcKO compared to WT mice. Thus, PEPCK activity, enzyme activity ratios and metabolite levels are consistent with an increase in gluconeogenesis in ShcKO liver.

The present study also found some evidence of changes in amino acid and lipid metabolism in the ShcKO mice. Consistent with increased use of amino acids for gluconeogenesis, the activities of tryptophan and tyrosine transaminase were increased in ShcKO compared to WT livers. Similar to our previous study which indicated increased ketogenesis in the ShcKO mice [9], total ketone body levels were higher in ShcKO compared to WT liver. This is likely a consequence of the higher β-oxidation of fatty acids as reported previously [9] that results in higher levels of acetyl-CoA. These changes in metabolism are expected in an animal that has shifted from glucose catabolism to gluconeogenesis and lipid catabolism.

**Fig. 9:**
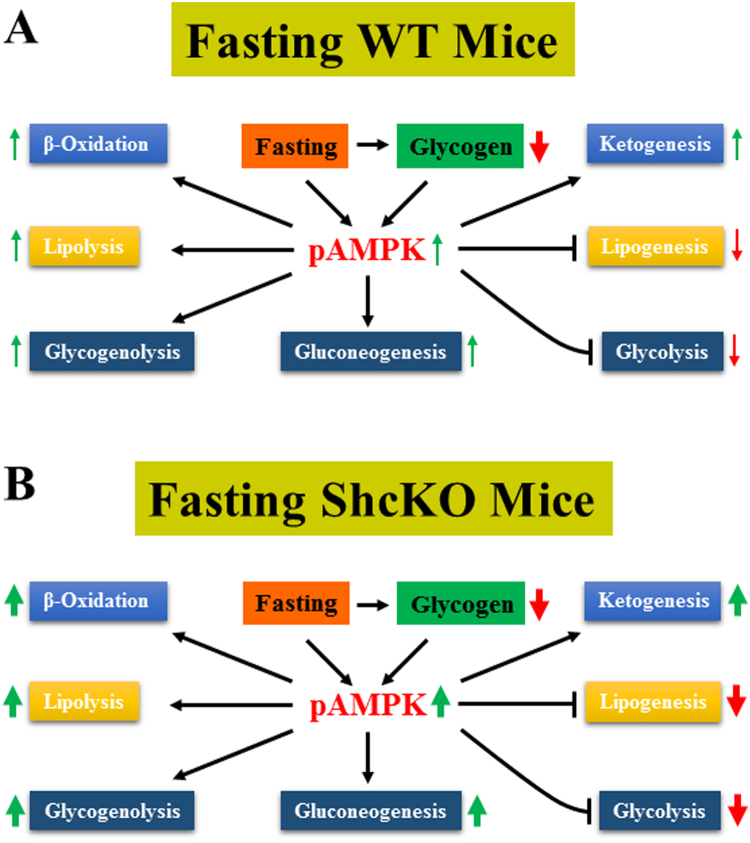
Schematic representation of the role of AMPK in the regulation of various pathways in WT (A) and ShcKO (B) mice in the fasted state. Up (green) and down (red) arrows indicate increased or decreased activities, respectively, of a given pathway/enzyme/metabolite when compared with their corresponding fed state. Thickness of the up or down arrows indicates the difference in the magnitude of increased or decreased activities in the fasted state when comparison is made between the fasted WT and ShcKO mice.

The transcription factors, AMPK and FOXO1, which play a role in transitioning metabolism toward gluconeogenesis and fatty acid metabolism were studied to see if they contributed to the changes in metabolism observed in the ShcKO mice. The present study does not find evidence that FOXO1 is contributing to the differences in metabolism between the ShcKO and WT mice, since FOXO1 levels did not differ between the two genotypes. However, it is possible that AMPK contributes to changes in metabolism observed in the ShcKO versus WT mice. AMPK is considered the major cellular energy sensor that is activated under conditions of metabolic/oxidative stress and nutritional deprivation and phosphorylated AMPK (pAMPK, activated) inhibits pathways that consume ATP, such as sterol and fatty acid synthesis, and activates pathways such as fatty acid oxidation that generate ATP [39], [40]. The role AMPK plays in the regulation of the various metabolic pathways investigated in the present study are summarized in Fig. 9. Activation of AMPK has been shown to suppress lipogenesis [41], inhibit pyruvate kinase and increase ketogenesis [42]. These changes in metabolism with activation of AMPK are similar to the shifts in metabolism observed in ShcKO mice. Interestingly, glycogen has also been reported to bind to AMPK and inhibit its action, therefore, playing a role in its regulation [43], [44], [45], [46]. The lower glycogen levels observed in this study in the fasting state may contribute to the activation of AMPK, specifically in the ShcKO mice. The display of higher pAMPK levels in the ShcKO versus WT mice under fasting conditions could contribute to shifts in metabolism in ShcKO mice. However, it remains to be determined how lower Shc protein levels are linked to phosphorylation of AMPK.

At the present stage of study, it is not possible to determine which specific Shc protein is responsible for the observed changes in metabolism since all three Shc proteins are decreased in livers from the ShcKO mouse. However, the current study demonstrates the important role Shc proteins play in metabolism, and future development of mouse models with controlled expression of individual Shc proteins will be needed to determine the role each Shc isoform plays in metabolism. Moreover, future work is needed to determine the rates of glycolysis and gluconeogenesis so as to establish flux through these two critical pathways under various nutritional conditions.

The results of the study show that decreased levels of Shc proteins lead to a shift in liver metabolism toward decreased glycolysis and increased gluconeogenesis and lipid metabolism. This result expands previous work demonstrating decreased glycolytic enzyme activity in skeletal muscle [8] and increased activities of enzymes involved in fatty acid and ketone body metabolism in liver and skeletal muscle [9] from ShcKO compared to WT mice, and indicates that Shc proteins should be considered as regulators of fuel selection.

## Appendix A Supplementary material

Supplementary materialSupplementary material

.

Supplementary materialSupplementary material

.

Supplementary materialSupplementary material

.

Supplementary materialSupplementary material

.

Supplementary materialSupplementary material

.

Supplementary materialSupplementary material
